# Risk factors for positive follow-up blood cultures in critically ill adults with Gram-negative bacteraemia

**DOI:** 10.1093/jacamr/dlae144

**Published:** 2024-09-10

**Authors:** Rebecca A Mayes, Emily A Siegrist, Julia Mathew, Stephen B Neely, Cindy B McCloskey, Bryan P White

**Affiliations:** University of Oklahoma Medical Center at OU Health, Department of Pharmacy, Oklahoma City, OK, USA; University of Oklahoma Medical Center at OU Health, Department of Pharmacy, Oklahoma City, OK, USA; University of Oklahoma Medical Center at OU Health, Department of Pharmacy, Oklahoma City, OK, USA; The University of Oklahoma College of Pharmacy, Pharmacy office of the Dean, Oklahoma City, OK, USA; The University of Oklahoma College of Medicine, Department of Pathology, Oklahoma City, OK, USA; University of Oklahoma Medical Center at OU Health, Department of Pharmacy, Oklahoma City, OK, USA

## Abstract

**Objectives:**

To evaluate the utility of follow-up blood cultures (FUBCs) for Gram-negative bloodstream infection (BSI) in ICU patients and identify risk factors for repeat positive cultures.

**Methods:**

This was a single-centre, retrospective cohort study of critically ill adults with Gram-negative BSI between 1 January 2015 and 1 January 2020. Critically ill patients with one or more blood cultures positive for a Gram-negative organism were included. Descriptive and inferential statistics were performed with an alpha of 0.05.

**Results:**

A total of 148 critically ill patients with Gram-negative BSI were included, with 42 patients (28.4%) having one or more positive FUBCs. The majority (66.2%) were admitted to a medical ICU. The most common organisms isolated were *Escherichia coli* (*n* = 56, 37.8%) and *Klebsiella pneumoniae* (*n* = 26, 17.6%). Significant patient risk factors associated with a positive FUBC on univariate regression included: MDR organisms, immunocompromised status, fever, vasopressor use at time of FUBC, lack of source control attainment, and higher quick Pitt bacteraemia score. Multivariable penalized logistic regression indicated that lack of source control containment and less time from index to FUBC remained significantly associated with repeat positive FUBC.

**Conclusions:**

This is the first study to investigate the use of FUBC for Gram-negative BSI in exclusively ICU patients. Risk factors for repeat positive FUBC in this population include lack of source control and less time between index and FUBC. Prospective studies are needed to fully elucidate the role of FUBCs in critically ill patients with Gram-negative BSI.

## Introduction

The practice of obtaining follow-up blood cultures (FUBCs) is standard of care in patients with *Staphylococcus aureus* bloodstream infections (BSIs) or candidaemia, in which duration of therapy is determined by the first negative blood culture to guide if further investigation is needed into untreated sites of infection.^1,2^ In *S. aureus* BSI and candidaemia, guidelines recommend obtaining repeat cultures every 1–4 days until the first negative culture is achieved.^1,2^ Likewise, previous studies have shown that between 17.6% and 86% of patients with Gram-negative BSI receive at least one FUBC; however, the utility of repeating cultures in this setting remains controversial as Gram-negative BSIs rarely have metastatic sites of infection.^3–5^ Positive FUBCs in patients with Gram-negative BSI have been identified as a marker of increased all-cause and attributable mortality, but most studies have found a low yield of positive repeat cultures in this setting. A recent guidance document does not recommend repeating blood cultures in the setting of uncomplicated Gram-negative BSI in clinically stable patients.^6–8^ Several risk factors have been associated with repeat positive cultures in the setting of Gram-negative BSI, including intravascular devices,^5,9^ MDR isolates,^5,9^ end-stage renal disease (ESRD) on haemodialysis (HD)^9^ and delay to appropriate antibiotics.^10^ Notably, these studies included few critically ill patients. Spaziante and colleagues^11^ conducted a retrospective review of 69 critically ill patients with Gram-negative BSI and found no difference in mortality in those with and without positive FUBC; however, patient-specific risk factors for positive follow-up cultures were not evaluated.

To date, no study has assessed the utility of FUBC in exclusively ICU patients with Gram-negative BSIs. Studies that have identified patient-specific risk factors for repeat positive cultures included few critically ill patients. There remains little guidance for clinicians on the appropriate use of repeat blood cultures in the ICU population; however, unnecessary blood cultures increase hospital length of stay and duration of antibiotic treatment.^12^

The purpose of this study was to explore the role of repeat blood cultures for critically ill patients at a single academic medical centre. Specifically, risk factors for repeat positive blood cultures were assessed to elucidate populations in which repeat cultures may be indicated and clinically valuable to improve diagnostic stewardship. Additionally, this study aimed to identify the incidence of positive repeat cultures in the critically ill population.

## Methods

This was a retrospective cohort study of microbiological data and patient medical records. Patients were identified using blood culture data obtained from the Vigilanz database (VigiLanz Corporation, Minneapolis, MN, USA). All ICU patients with a positive blood culture for a Gram-negative organism, excluding obligate anaerobic organisms, between 1 January 2015 and 1 January 2020 were included for initial review. Standard adult blood cultures were collected and incubated in BD BACTEC™ Plus Aerobic medium in plastic culture vials and BD BACTEC™ Lytic Anaerobic medium in plastic culture vials. Vials were incubated in the BD BACTEC™ FX Blood Culture System until positive or for a total of 5 days if negative. Patients were included if they received at least one FUBC within 24–96 h of index culture. Exclusion criteria included death within 24 h of index culture, polymicrobial index cultures excluding contamination, or incomplete medical records.

Patient-specific data were collected using the electronic medical record and Meditech^®^ (Medical Information Technology, Inc., Westwood, MA, USA). Blood culture data were collected and included the organism identified and antimicrobial resistance (e.g. ESBL-producing) if applicable. This information was collected for index culture and all repeat blood cultures performed within 24–96 h of index culture and it was noted if a different Gram-negative organism was identified upon repeat culture.

Demographic data were collected and included: age, sex and ICU service (e.g. trauma, medical). The patient-specific data that were obtained included: quick Pitt bacteraemia score,^13^ fever, source of infection, immunosuppression, intravascular catheters, cardiac devices, urinary hardware, haemodialysis dependence, continuous renal replacement therapy, time from admission to index culture (days), time to appropriate antibiotics (hours), duration of antibiotic treatment (days), source control achieved before FUBC (yes or no), hospital length of stay (days), and in-hospital all-cause mortality. All data points were collected at time of index culture. Fever and vasopressor use were collected at time of index and follow-up blood culture(s). Time to appropriate antibiotics was calculated in hours, as time from laboratory receipt of index blood culture to time of first administration of an active antibiotic. In patients with multiple episodes of Gram-negative BSI requiring multiple hospitalizations during the study period, only the first hospitalization was included in the analysis.

### Definitions

An FUBC was defined as at least one bottle of blood culture within 24–96 h of index blood culture. FUBCs were considered positive if the same Gram-negative organism as the index culture was identified. Immunosuppression was defined as meeting one of the following criteria: corticosteroid therapy equivalent to prednisone ≥2 mg/kg or ≥20 mg daily for at least 14 days; biological agents in the preceding 30 days; solid organ transplant; haematopoietic stem cell transplant in the preceding 1 year; cancer chemotherapy within 6 months; congenital immunodeficiency; and HIV with CD4 count ≤200 cells/mm.^3,14^ Fever was defined as one or more documented temperatures ≥38.3°C in the 24 h preceding index or FUBC.^15^ A patient was considered to have achieved source control if one of the following was met: (i) urinary tract: exchange or removal of urinary catheter(s) or nephrostomy tube(s), removal of nephrolithiasis or other obstruction; (ii) intra-abdominal: drainage of abscesses/collection, debridement of infected necrotic tissues, removal of infected mesh; (iii) skin and soft tissue: incision and drainage of abscess, debridement of the infected/necrotic tissue or amputation; (iv) central venous catheter: removal and/or exchange of vascular catheter.^16^ Appropriate antibiotics were considered to be any antibiotic listed as susceptible on susceptibility report. Refer to Appendix 1 (available as Supplementary data at *JAC-AMR* Online) for definitions of MDR organisms.

### Statistical analysis

Cases were defined as patients with one or more positive FUBCs within 24–96 h of index culture, and controls were those with negative FUBC. Bivariate analysis was performed to examine potential risk factors for case status. Data were summarized using descriptive statistics. Categorical variables are reported as frequency (percentage) and groups were compared using χ^2^ tests or exact equivalent if expected cell sizes were small. Continuous variables are reported as mean (SD) or median (IQR) depending on skew of data. To compare case/control groups we used *t*-tests or Mann–Whitney *U* tests.

For the primary objective, a logistic regression analysis was performed to identify risk factors for a positive FUBC with the same Gram-negative organism. Both patient-specific and hospital-specific variables were evaluated for inclusion in the adjusted model. Variables of interest were considered for backward selection in the multivariable analysis if *P* < 0.2 and retained if *P* < 0.1; however, time from index to appropriate antibiotic and time from index to FUBC were planned to be included regardless. A penalized logistic regression with Firth correction was used to account for complete separation observed within the source control variable. Unadjusted and adjusted ORs and 95% CIs are reported.

The yield of positive FUBCs was calculated by dividing the number of cases found to be positive by the number of total episodes of Gram-negative BSI that had repeat cultures performed. The yields of FUBCs in patients with and without independent risk factors identified in the multivariate analysis were compared. SAS software 9.4 (SAS Institute Inc., Cary, NC, USA) was used for all analyses. Alpha was set at 0.05.

## Results

A total of 399 patients with Gram-negative BSI between January 2015 and January 2020 were included for initial review. Of those, 74, 70 and 19 patients were excluded due to polymicrobial index cultures, death within 24 h, or lack of admission to a critical care service, respectively. Additionally, 80 patients were excluded for not having FUBC drawn, and 8 were excluded for incomplete records. A total of 148 patients met the inclusion criteria, with 42 (28.4%) having positive FUBC and 106 (71.6%) with negative FUBC.

The majority of patients were in the medical ICU (*n* = 98, 66.2%), followed by trauma (*n* = 19, 12.8%) and surgical (*n* = 15, 10.1%) ICUs. The median (IQR) age was 59 years (48–69), and the majority were male (*n* = 82, 55.4%). The most common organisms isolated were *Escherichia coli* (*n* = 56, 37.8%), *Klebsiella pneumoniae* (*n* = 26, 17.6%), *Enterobacter cloacae* (*n* = 13, 8.8%) and *Pseudomonas* species (*n* = 13, 8.8%). The most common source of infection was urinary (*n* = 45, 30.4%), followed by respiratory (*n* = 32, 21.6%) and intra-abdominal (*n* = 31, 20.9%) sources. Two patients were on mechanical circulatory support (i.e. extracorporeal membrane oxygenation), and 24 (16.2%) were on HD or renal replacement therapy at time of FUBC. Four patients had cardiac devices or hardware: two with permanent pacemakers and two with bioprosthetic valves. The primary sources of infection identified in these patients were respiratory (*n* = 2), urinary (*n* = 1) and central venous catheter (*n* = 1). None of these patients had positive FUBC or required removal of the cardiac device or hardware. The overall rate of MDR organisms was 20.9% (*n* = 31), with the majority being extended-spectrum cephalosporin-resistant organisms (*n* = 24, 16.2%). Additional MDR organisms isolated were carbapenem-resistant Enterobacteriaceae (*n* = 3), carbapenem non-susceptible *Acinetobacter* spp. (*n* = 2), MDR *Pseudomonas* spp. (*n* = 1) and carbapenem non-susceptible *Pseudomonas* spp. (*n* = 1).

FUBCs were obtained a median (IQR) of 41.6 h (32.7–51.7) following index culture. Patients with positive FUBC had repeat cultures drawn significantly earlier than those with negative FUBC [35.2 (28.2–42.0) versus 44.2 (33.2–55.4) h; *P* = 0.0013]. There was no difference in hospital mortality between patients with and without positive FUBC (45.2% versus 38.7%, *P* = 0.4637). Additionally, duration of antimicrobial therapy did not differ between groups and this remained non-significant when accounting for patients who died during hospitalization. Characteristics of patients with and without positive FUBC are described in Table 1.

**Table 1.** Characteristics of patients with and without positive FUBC

Characteristic	Positive FUBC (*n* = 42)	Negative FUBC (*n* = 106)	*P* value
Median age, y (IQR)	60.5 (44.0–69.0)	59.0 (49.0–69.0)	0.8152
Male, *n* (%)	23 (54.8)	59 (55.7)	0.921
Medical ICU, *n* (%) Non-medical ICU, *n* (%)	31 (73.8) 11 (26.2)	67 (63.2) 39 (36.8)	0.2189
Median quick Pitt bacteraemia score (IQR)	3 (2–3)	2 (1–3)	**0**.**0425**
Haemodialysis or renal replacement therapy, *n* (%)	5 (11.9)	19 (17.9)	0.3704
Organism, *n* (%)			0.672
* E. coli*	18 (42.9)	38 (35.8)	
* K. pneumoniae*	9 (21.4)	17 (16.0)	
* E. cloacae*	3 (7.1)	10 (9.4)	
* Pseudomonas* spp.	4 (9.5)	9 (8.5)	
MDR, *n* (%)	14 (33.3)	17 (16)	**0**.**0197**
Non-lactose-fermenting bacteria^a^, *n* (%)	8 (19)	32 (30.2)	0.1688
Source, *n* (%)			0.791
* *Urinary	11 (26.2)	34 (32.1)	
* *Respiratory	7 (16.7)	25 (23.6)	
* *Intra-abdominal	12 (28.6)	19 (17.9)	
* *IV catheter	4 (9.5)	11 (10.4)	
* *Skin/soft tissue	1 (2.4)	4 (3.8)	
* *Other	2 (4.8)	3 (2.8)	
* *Unclear	5 (11.9)	10 (9.4)	
Immunocompromised, *n* (%)	16 (38.1)	21 (19.8)	**0**.**0206**
Cardiac device or hardware, *n* (%)	0 (0)	4 (3.8)	0.578
Permanent pacemaker	0 (0)	2 (1.8)	—
Bioprosthetic valve	0 (0)	2 (1.8)	—
Median length of stay, d (IQR)	17.5 (9.0–33.0)	17.0 (8.0–28.0)	0.9474
Median time from index to FUBC, h (IQR)	35.2 (28.2–42.0)	44.2 (33.2–55.4)	**0**.**0013**
Median time to appropriate antibiotic, h (IQR)	14.0 (3.7–35.8), *n* = 33^b^	10.0 (1.0–30.6), *n* = 77^b^	0.1151
Fever, *n* (%)	18 (42.9)	24 (22.6)	**0**.**0139**
Vasopressor use, *n* (%)	25 (59.5)	36 (34)	**0**.**0044**
Source control achieved, *n* (%)	0 (0)	29 (27.4)	**0**.**0004**
Time from admission to index culture, h, median (IQR)	1.34 (0.525–11.35)	2.86 (0.66–11.695)	0.4749
Median duration of antimicrobial therapy, d (IQR)			
Overall	7.39 (3.21–15.50), *n* = 41^c^	7.72 (4.23–14.46), *n* = 106	0.8404
Among those who expired during stay	2.90 (0.96–7.39), *n* = 18	5.60 (3.30–12.30), *n* = 41	0.0997
Among those who did not expire during stay	13.51 (6.47–17.53), *n* = 23	9.45 (5.60–14.46), *n* = 65	0.1410

Bold values in RH column as presume they indicate statistical significance.

^a^Non-lactose-fermenting bacteria included: *Pseudomonas* spp., *Proteus* spp., *Acinetobacter* spp., *Burkholderia* spp., *Stenotrophomonas* spp*., Morganella morganii* and *Serratia* spp.

^b^Patients with appropriate antimicrobial administered after index blood culture received by microbiology laboratory. Patients with appropriate antimicrobial administration prior to blood culture drawn were not included.^17^

^c^One patient with MDR *Pseudomonas* bacteraemia did not receive an active antimicrobial prior to time of death.

Table 2 describes the univariate and multivariate regression analyses. On the univariate logistic regression analysis, significant patient-specific risk factors associated with a positive FUBC included: MDR organisms, immunocompromised status, fever, vasopressor use at time of FUBC, lack of source control attainment, and higher quick Pitt bacteraemia score. On the multivariable penalized logistic regression analysis, patients who did not attain source control had 25.2 times greater adjusted odds of a positive FUBC than those who did (*P* = 0.025). Patients with a fever at the time of the FUBC had two times greater adjusted odds of a positive FUBC (*P* = 0.0801); however, this was not statistically significant. Additionally, any +1 h between the index culture to FUBC resulted in 3% lower odds (*P* = 0.0249) of a positive repeat culture.

**Table 2.** Logistic regression model of patients with positive FUBC

Variable	Comparison	Reference group or mean (SD)	Univariate logistic regression models^a^		Multivariable penalized logistic regression model^b^	
			OR (95% CI)	*P* value	Adjusted OR (95% CI)	*P* value
MDR	Yes (21%)	No (79%)	2.62 (1.15–5.98)	0.0222		
Time to appropriate antibiotics, h	Any +1 h	Mean = 8.07	1.01 (0.99–1.02)	0.2377	1.01 (0.99–1.02)	0.3084
Time index to FUBC, h	Any +1 h	Mean = 44.2	0.95 (0.93–0.98)	0.0016	0.97 (0.94–1.00)	0.0249
Non-lactose-fermenting bacteria	Yes (27%)	No (73%)	0.54 (0.23–1.31)	0.1727		
Fever at time of FUBC	Yes (28%)	No (72%)	2.56 (1.20–5.49)	0.0155	2.08 (0.92–4.74)	0.0801
Vasopressor use at time of FUBC	Yes (41%)	No (59%)	2.86 (1.37–5.97)	0.0051		
Immunocompromised	Yes (25%)	No (75%)	2.49 (1.14–5.46)	0.0227		
Quick Pitt bacteraemia score	Any +1 point	Mean = 2.24	1.42 (1.01–2.00)	0.0447		
Source control achieved before FUBC	No/unknown (80%)	Yes (20%)	32.37 (1.84–570.00)	0.0175	25.21 (1.49–426.39)	0.0253

^a^Univariate models used traditional logistic regression except for source control, which required a penalized logistic regression model due to complete separation (i.e. no patient who achieved source control had a positive FUBC).

^b^All listed variables were considered for the multivariable model using backward selection (enter if *P* < 0.20 and retained if *P* < 0.1) with the exception of both time from index to appropriate antibiotic scan and time from index to FUBC, which were included regardless. The penalized logistic approach was used for the multivariable model to accommodate source control.

## Discussion

There is increasing discussion of the importance of diagnostic stewardship, particularly in critical care settings.^18^ Mitaka and colleagues^12^ found that patients with Gram-negative BSI who received one or more FUBCs had a longer length of stay and duration of antimicrobials. This study concluded that specific criteria must be identified to carefully select patients for whom repeat blood cultures have a high pretest probability. Our study aimed to identify those specific criteria in exclusively adult ICU patients.

The results of this study identified two important observations. Most significantly, subpopulations of critically ill patients were identified with a higher risk of positive FUBC. In the adjusted model, patients lacking source control at time of FUBC were more likely to have repeat positive cultures. Additionally, each +1 h between index and FUBC reduced the incidence of a repeat positive culture. There was a high incidence of positive FUBC compared with previous studies, with 28.4% of patients having one or more positive FUBCs. Overall, this study provides specific risk factors where FUBCs should be considered in Gram-negative BSI as opposed to being considered in all critically ill patients with Gram-negative BSI.^19^

Few studies have previously evaluated risk factors for repeat positive cultures in Gram-negative BSI. Canzoneri *et al*.^20^ explored rates of positive FUBC in patients with Gram-positive or Gram-negative bacteraemia. A total of 140 patients with FUBC obtained had Gram-negative bacteraemia, and 8 (5.7%) of those had a positive FUBC. Due to the small number of patients with Gram-negative infections, analyses to identify risk factors for positive FUBC could not be performed in this subgroup. Kang and colleagues^21^ assessed exclusively *K. pneumoniae* BSI and found that patients with higher Charlson comorbidity index, solid organ transplantation, intra-abdominal source and unfavourable treatment responses were associated with positive repeat cultures. In this study, 19.4% of patients were admitted to the ICU. Giannella *et al*.^3^ found that 38.5% of patients had one or more positive FUBCs but noted that those with FUBC were more severely ill patients. Additionally, Mitaka *et al*.^9^ identified ESRD on HD, presence of an intravascular device, and MDR organisms as independent risk factors for positive FUBC. Maskarinec *et al*.^6^ found that 20% of patients had repeat positive blood cultures, and those with persistently positive cultures were less likely to be on appropriate antimicrobials, more likely to have cardiac devices, be HD dependent or have an endovascular source. Nonetheless, these studies are limited by the small number of critically ill patients.

The percentage of patients with positive FUBC was slightly higher in our cohort than in previous studies. This could be because admission to an ICU in itself may be an independent risk factor for positive FUBC, as critically ill patients generally have more comorbidities and require more procedures or invasive devices.^9^ A few clinically relevant risk factors did not meet criteria for inclusion in the logistic regression model, potentially due to our small sample size. For example, numerically more patients with intra-abdominal infections had positive FUBC (28.6% versus 17.9%), but this did not meet statistical significance. As no patients had source control in the positive FUBC group, a large point estimate was produced with wide CIs even when using penalized logistic regression. Additionally, a recent meta-analysis found ESRD on HD to be a significant risk factor for repeat positive cultures; however, only 24 patients (16.2%) in our study met this criterion and it was not found to be significant.^22^ Furthermore, we only included FUBCs drawn between 24 and 96 h, which may have skewed the distribution of positive FUBC. Lastly, only four patients had cardiac devices or hardware and none were found to have positive FUBC; therefore, we cannot rule this out as a potential risk factor.

There are several limitations to this study. The study period of 2015–2020 excluded the COVID pandemic, and these results may not apply to critically ill patients with COVID. Likewise, most patients were in the medical ICU, limiting the generalizability to surgical populations. Due to the retrospective design, we are not aware of the reason providers ordered repeat blood cultures. It is possible that those with FUBCs drawn were perceived to have a higher severity of illness compared with those who did not, putting them at higher risk of a positive FUBC. Data on the specific type of source control procedure or duration of bacteraemia were not collected. Without data on the duration of bacteraemia, these data may not apply to patients with septic thrombosis where the duration of bacteraemia is often prolonged.^23^ Additionally, this study has the usual limitations of observational studies, including the inability to adjust for unknown confounders.

In conclusion, these data identify risk factors for positive FUBCs in exclusively critically ill patients with Gram-negative BSI. Critically ill patients with lack of source control may be more likely to have positive FUBCs. As increased time to FUBC was associated with reduced incidence of positive FUBC, clinicians may consider waiting 48 h to repeat blood cultures, if desired, for most patients other than those with suspected endocarditis.^24^ Although there are multiple studies questioning the utility of repeat blood cultures in uncomplicated Gram-negative BSI, our data raise the question whether this applies to critically ill patients. Prospective studies are needed to confirm these results.

## Supplementary Material

dlae144_Supplementary_Data

## Supplementary data

Appendix S1 is available as Supplementary data at *JAC-AMR* Online.
