# Supplementary material for: Risk factors for positive follow-up blood cultures in critically ill adults with Gram-negative bacteraemia
Source: JAC Antimicrob Resist. 2024 Sep 10;6(5):dlae144. doi: 10.1093/jacamr/dlae144 (PMC11384144; doi:10.1093/jacamr/dlae144)
Supplement: dlae144_Supplementary_Data [file dlae144_supplementary_data.docx]

Appendix 1. Definitions of multi-drug resistant organisms.^[[1]](#footnote-1)^

| Carbapenem-resistant Enterobacteriaceae | Any *Citrobacter amalonaticus*, *Citrobacter freundii, Citrobacter koseri, Enterobacter* spp., *E. coli*, *Klebsiella aerogenes*, *Klebsiella oxytoca*, *Klebsiella pneumoniae*, and *Serratia marcescens* that has tested Resistant (R) to at least one of the following: imipenem, meropenem, doripenem, or ertapenem OR any *Proteus mirabilis*, *Proteus penneri*, *Proteus vulgaris*, and *Morganella morganii* that has tested Resistant (R) to at least one of the following: meropenem, doripenem, or ertapenem | |
| --- | --- | --- |
| Carbapenem-nonsusceptible | *Pseudomonas aeruginosa* that has tested either Intermediate (I) or Resistant (R) to at least one of the following: imipenem, meropenem, or doripenem | |
| Extended-spectrum cephalosporin-resistant E.coli | Any *E. coli* that has tested Resistant (R) to at least one of the following: cefepime, ceftriaxone, cefotaxime, or ceftazidime | |
| Extended-spectrum cephalosporin-resistant *Klebsiella pneumoniae*/*oxytoca* | Any *Klebsiella oxytoca* or *Klebsiella pneumoniae* that has tested Resistant (R) to at least one of the following: cefepime, ceftriaxone, cefotaxime, or ceftazidime. | |
| Multidrug-resistant Pseudomonas aeruginosa | *Pseudomonas aeruginosa* that has tested either Intermediate (I) or Resistant (R) to at least one drug in at least three of the following five categories: 1. Extended-spectrum cephalosporin (cefepime, ceftazidime) 2. Fluoroquinolones (ciprofloxacin, levofloxacin) 3. Aminoglycosides (amikacin, gentamicin, tobramycin) 4. Carbapenems (imipenem, meropenem, doripenem) 5. Piperacillin/tazobactam | |
| Carbapenem-nonsusceptible Acinetobacter spp. | Any Acinetobacter spp. that has tested either Intermediate (I) or Resistant (R) to at least one of the following: imipenem, meropenem, or doripenem | |
| Multidrug-resistant Acinetobacter spp. | Any Acinetobacter spp. that has tested either Intermediate (I) or Resistant (R) to at least one drug in at least three of the following six categories: 1. Extended-spectrum cephalosporin (cefepime, ceftazidime, ceftriaxone, cefotaxime) 2. Fluoroquinolones (ciprofloxacin, levofloxacin) 3. Aminoglycosides (amikacin, gentamicin, tobramycin) 4. Carbapenems (imipenem, meropenem, doripenem) 5. Piperacillin/tazobactam 6. Ampicillin/sulbactam |  |

1. Antimicrobial-Resistant Phenotype Definitions. <https://www.cdc.gov/nhsn/pdfs/ps-analysis-resources/phenotype_definitions.pdf>. [↑](#footnote-ref-1)
